# An ethnobotanical survey of plants used to manage HIV/AIDS opportunistic infections in Katima Mulilo, Caprivi region, Namibia

**DOI:** 10.1186/1746-4269-6-25

**Published:** 2010-09-11

**Authors:** Kazhila C Chinsembu, Marius Hedimbi

**Affiliations:** 1 University of Namibia, Faculty of Science, Department of Biological Sciences, P/B 13301, Windhoek, Namibia

## Abstract

Katima Mulilo has the highest burden of HIV/AIDS in Namibia. Due to several constraints of the antiretroviral therapy programme, HIV-infected persons still use ethnomedicines to manage AIDS-related opportunistic infections. Despite the reliance on plants to manage HIV/AIDS in Katima Mulilo, there have been no empirical studies to document the specific plant species used by traditional healers to treat AIDS-related opportunistic infections. In this study, an ethnobotanical survey was conducted to record the various plant families, species, and plant parts used to manage different HIV/AIDS-related opportunistic infections in Katima Mulilo, Caprivi region, Namibia. The results showed that a total of 71 plant species from 28 families, mostly the Combretaceae (14%), Anacardiaceae (8%), Mimosaceae (8%), and Ebanaceae (7%), were used to treat conditions such as herpes zoster, diarrhoea, coughing, malaria, meningitis, and tuberculosis. The most plant parts used were leaves (33%), bark (32%), and roots (28%) while the least used plant parts were fruits/seeds (4%). Further research is needed to isolate the plants' active chemical compounds and understand their modes of action.

## Background

The first case of Acquired Immunodeficiency Syndrome (AIDS) in Namibia was identified in 1986 [1]. Since then, Human Immunodeficiency Virus (HIV) infection has spread rapidly throughout the country. From the first sentinel surveillance survey in 1992 when the HIV prevalence rate was 4.2%, the epidemic rose to15.4% in 1996 and peaked in 2002 at 22.0%, before declining to 19.7% in 2004, and 17.8% in 2008 [1]. Now, the country has a generalized HIV/AIDS epidemic with about 230,000 to 250,000 people living with HIV/AIDS [2,3]. HIV prevalence rates among Namibians aged 15-49 years were estimated at 12.4-18.1%, with an annual death rate of about 7,100 attributable to AIDS [2]. Namibia also has one of the highest tuberculosis infection rates in the world, with 63.5% of tuberculosis cases being HIV positive [3]. Given that Namibia has a total population of about 2 million people, these grim statistics have put Namibia in the top five of the most HIV/AIDS-burdened countries in the world [4,5].

Out of Namibia's 13 political regions, the Caprivi region is the hardest hit by HIV/AIDS. In 2008, the HIV prevalence rate among pregnant women was 31.7% in Katima Mulilo, the capital of the Caprivi region, while it was 13.1% in Gobabis (in the Omaheke region) and 21.7% in Windhoek (Khomas region) [4]. HIV prevalence rates in Katima Mulilo rose from 14% in 1992, to 25% in 1994, 29% in 1998, 43% in 2002, and 39.4% in 2006 [1]. Among pregnant women aged 15-24 years, HIV prevalence rates were 38.9% in 2004, 30.9% in 2006, and 24.1% in 2008; while among those aged 25-49 years, the HIV prevalence rates were 47.4% in 2004, 49.4% in 2006, and 40.3% in 2008 [1].

A confluence of geopolitical, biological, socio-economic, behavioural, and cultural factors is working to make Katima Mulilo one of the worst HIV epidemics in Southern Africa [4]. Katima Mulilo is situated at a major international border that links five countries: Angola, Botswana, Namibia, Zambia, and Zimbabwe. The Trans-Caprivi highway passes through Katima Mulilo, bringing heavy traffic to and from Southern Africa. Truckers, merchants, and migrant workers are serviced by a booming commercial sex industry at the border town of Katima Mulilo [4]. Other factors that have silently conspired to fuel the HIV/AIDS epidemic in Katima Mulilo are: low frequency of circumcision, high levels of poverty, low levels of condom use, early sexual debut, multiple sex partners, and strong beliefs in witchcraft [4,6]. For example, many inhabitants of Katima Mulilo believe that HIV/AIDS is spread through *mulaleka*, a witchcraft practice believed to make someone have forced sex with another person by remote [6]. Such beliefs subtract from HIV/AIDS prevention and treatment.

On the other hand, the Lozi people of Katima Mulilo (generally known as Caprivians) have very strong beliefs in the use and efficacy of ethnomedicines. Although most HIV/AIDS-infected people that need treatment can access antiretroviral therapy (ART) from local hospitals and health centres, several constraints of the ART program compel many HIV-infected Caprivians to use herbal plants to manage HIV/AIDS-related opportunistic infections [6]. Others use herbal plants to offset side-effects from ART. Despite the strong anecdotal evidence regarding the traditional uses of plants to manage HIV/AIDS in the Caprivi region, there have been no empirical studies to pinpoint the specific plant species used by traditional healers to treat AIDS-related opportunistic infections. Documentation of anti-HIV plant species will help preserve this important indigenous knowledge resource, and may also lead to the isolation of novel chemical compounds that can be developed into newer antiretroviral drugs. Therefore, this paper is an inaugural and modest attempt to ethnobotanically survey and record the various plant species used to manage HIV/AIDS-related opportunistic infections in Katima Mulilo, Caprivi region, Namibia.

## Methods

### Study site

**Figure 1:**
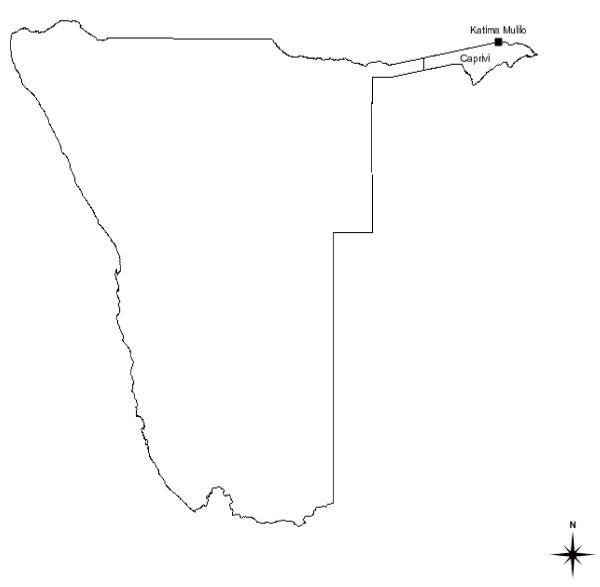
**Map of Namibia showing Katima Mulilo town in the Caprivi region**.

The study was carried out in Katima Mulilo, regional administrative capital of the Caprivi region (Fig. 1). Caprivi is one of the 13 regions of Namibia and takes its name from the Caprivi Strip. Popularly known as the 'arm' of Namibia, the Caprivi is a semi-tropical region that lies north-east of the country. It is a major transit point that borders Angola, Botswana, Zambia, and Zimbabwe. In the northwest, it borders the Cuando Cubango province of Angola. In the north, it borders the western province of Zambia, while in the south it borders Botswana. Therefore, the Caprivi is almost entirely surrounded by foreign countries. Its only domestic border is a short connection to the west with the Okavango region of Namibia. The small town of Katima Mulilo forms a crossing point served by the Trans-Caprivi highway from Walvis Bay and Windhoek. The highway provides the main transport route to south-east Angola, northern Botswana and western Zambia.

The Caprivi area experiences high temperatures and is the wettest region of Namibia. The Caprivi receives heavy rains during the rainy season from December to March, and has high temperatures throughout the year, though winter nights are cold. Its terrain is well vegetated, mostly made up of swamps, floodplains, wetlands, and deciduous woodlands dominated by trees such as the Zambezi teak. Most of Katima Mulilo is inhabited by the Lozi ethnic group who also live in western Zambia, northwest Zimbabwe, and northern Botswana. According to National Population and Housing Census projections of 2001, the Caprivi region has a total population of 87,058 people [7]. The relative socioeconomic situation in the region compares poorly to other parts of the country [7].

### Data collection

Snow-ball sampling was applied in this study with traditional healers, the main informants in the survey, being identified by the regional HIV/AIDS coordinator and the chairperson of the local traditional healers' association. A total of 14 traditional healers were interviewed in June and November 2009, and April 2010. The age of respondents ranged from 52-78 years, and 73% of the respondents were male. The regional HIV/AIDS coordinator was the English-Lozi translator during the conversations between the healers and the research team. After explaining the objectives of the research and seeking their consent, the traditional healers were engaged in a semi-structured interview. During the conversations, data on the local names of plants and plant parts used to treat various opportunistic infections related to HIV/AIDS were recorded. Traditional healers were used as guides during field trips to collect plant voucher specimens which were later identified at the University of Namibia.

## Results

**Figure 2:**
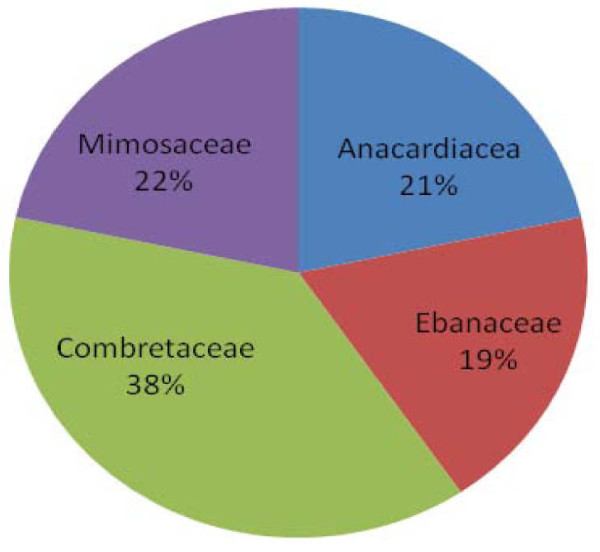
**Percentage use of plant families**.

**Figure 3:**
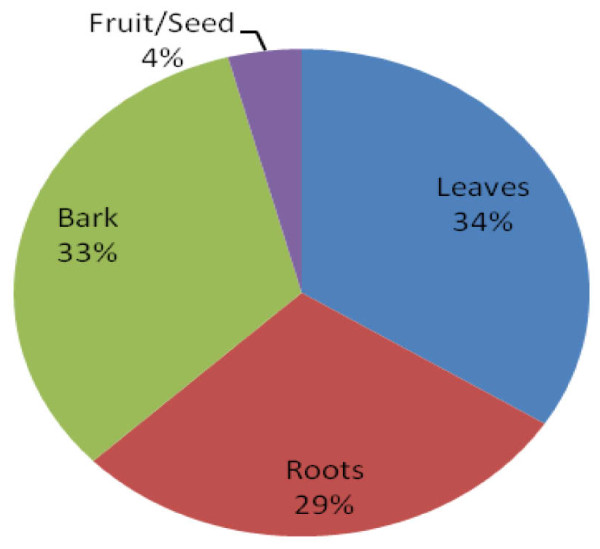
**Percentage of plant parts used**.

**Figure 4:**
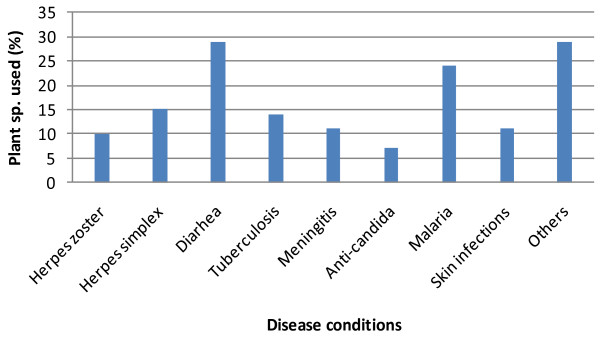
**Percentage use of plants to treat disease conditions**.

A total of 71 plants from 28 families were identified (Table 1). The most used families were Combretaceae (14%), Anacardiaceae (8%), Mimosaceae (8%), and Ebanaceae (7%) (Fig. 2). The most plant parts used were leaves (33%), bark (32%), and roots (28%) (Fig. 3). The least used plant parts were fruits/seeds (4%). The proportions of plant species used to treat various conditions were: diarrhoea (29%), malaria (24%), herpes simplex 15%, tuberculosis (14%), meningitis (11%), skin infections (11%), herpes zoster 10%, candidiasis (7%), and others (29%) (Fig. 4).

**Table 1:** Plants that are used to treat HIV/AIDS related disease conditions in Katima Mulilo, Caprivi region, Namibia

Family	Collection No.	Scientific name	Common name	Local name	Parts used	Disease conditions treated	Mode of application
Aloaceae	CM15	*Aloe zebrina*	Aloe	Chiforoforo	Leaves	Herpes zoster	Rubbing
Anacardiaceae	CM09	*Sclerocarya birrea *(A. Rich) Hochst	Marula	Mulula	Root bark	Candidiasis Diarrhoea	Rubbing Drinking
	CM35	*Lannea stuhlmannii *Engl.	False Marula	Rungomba	Roots	Herpes zoster, Herpes simplex, Skin infections,	Rubbing Rubbing
	CM54	*Rhus natalensis *Krauss		Rungomba	Leaves/Roots	Cryptococcal meningitis,	Drinking
	CM10	*Lannea schimperi *(A. Rich) Engl.		Kangawa	Bark	Tuberculosis,	Drinking
	CM55	*Lannea zastrowiana*		Rungomba		Skin rashes, Herpes zoster, Herpes simplex,	Rubbing
	CM36	*Rhus tenuinervis*			Bark	Chronic diarrhoea	Drinking

Annonaceae	CM37	*Xylopia *spp		Situnduwanga Malolo	-	Stomachache, Malaria	Drinking Drinking
	CM56	*Annona senegalensis *Pers.	Dwarf custard apple	Malolo	Root	Herpes zoster, Cryptococcal meningitis,	Rubbing Drinking
	CM65	*Annona stenophylla*				Skin infections	Rubbing

Bignonaceae	CM07	*Kigelia africana *(Lam.) Benth.	Sausage tree	Mupolota	Bark/Fruit	Herpes simplex, diarrhoea	Rubbing Drinking

Bombaceae	CM34	*Adansonia digitata *L.	Baobab	Mubuyu	Leaves, Bark, Roots	Malaria, Dysentery Diarrhoea	Drinking, Steaming Drinking Drinking

Burseraceae	CM57	*Commiphora africana*	-	Mubobo	Roots	Swollen pancreas	Drinking

Capparaceae	CM11	*Capparis erythrocarpos *Isert.		Ntulwantulwa -	Roots	Skin rashes, Tuberculosis,	Rubbing Drinking
	CM53	*C. tomentosa*			Roots	Cryptococcal meningitis, Oral candidiasis, Herpes zoster, Herpes simplex, Chronic diarrhoea	Drinking Chewing, Oral wash Rubbing Drinking

Chrysobalanaceae	CM02	*Parinari curatellifolia *Benth.	Mobola Plum	Mubula	Bark and Root	Skin rashes, herpes zoster, herpes simplex, Tuberculosis, Chronic diarrhoea,	Rubbing Drinking Drinking

Clusiaceae	CM12 CM32	*Garcinia buchananii *Bak. *G. livingstonei*	African Mongosteen	Mukononga	Bark/Root	Cryptococcal meningitis, Herpes zoster, Herpes simplex, Skin rashes Tuberculosis Chronic diarrhoea,	Drinking Rubbing Rubbing Drinking Drinking

Combretaceae	CM08	*Combretum glutinosum*		Mububu Muzwili	Leaves	Malaria, diarrhoea	Steaming, Drinking
	CM52	*C. latialatum*,				Malaria, diarrhoea	Steaming, Drinking
	CM31	*C. micranthum*,				Malaria, diarrhoea	Steaming, Drinking
	CM58	*C. platysterum*,				Malaria, diarrhoea	Steaming, Drinking
	CM38	*C. spinesis*.				Malaria, diarrhoea	Steaming, Drinking
	CM66	*C. collinum *Sound.	Weeping	Mububu	Leaves,	Chronic diarrhoea,	Drinking
	CM30	*Terminalia mollis *Laws	bushwillow Kudu Bush	Muhonono	Bark. Roots Bark	tuberculosis Cryptococcal	Drinking Drinking
	CM67	*T. sericea*,	Mubeziyam	Mukenge		meningitis,	
	CM47	*C. apiculatum*,					
	CM18	*C. alaeagnoides*	pampa			Tuberculosis, Diarhoea	Drinking

Cucurbitaceae	CM06	*Cucumis culeatus *Cogn.		Katende Konnsa	Root	Malaria	Steaming

Ebanaceae	CM29	*Diospyros mespiliformis*	Jackal Berry,	Muchenje	Bark and Leaves	Malaria	Steaming
	CM28	*Diospyros melanoxylon*,	African Ebony	Mujongoro	Leaves and bark	Malaria	Steaming
	CM39	*D. peregrina*,					
	CM51	*D. sylvatica*,					
	CM59	*D. tomentosa*					

Euphorbiaceae	CM27 CM40	*Croton lechleri *Müll. Arg. *Antidesma venosum *Tul.	Tassel berry	Mukena -	Bark Roots	Diarrhoea, lack of appetite, anaemia Tuberculosis, chronic diarrhoea, Oral candidiasis	Drinking Chewing, Oral wash

Fabaceae	CM50	*Dichrostachys cinerea *(L.) Wight & Arn	-	Muselesele	Leaves	Oral candidiasis	Chewing, Oral wash

Leguminosae	CM05 CM60	*Guibourtia tessmannii* *Pterocarpus erinaceus*		Muzauli Mulombe	Bark Leaves, Stem	Malaria Dysentery, diarrhoea	Drinking Drinking

Malvaceae	CM13 CM68	*Hibiscus fuscus *Garcke *H. sabdariffa*		Sindambi	Leaves	Chronic diarrhoea	Drinking

Mimosaceae	CM71	*Albizia amara *(Roxb.) Boiv.			Leaves	Stomach pains	Drinking
	CM41	*A. anthelmintica* Brong.	Camelthorn	Muhoto Mikakanyi Mukotokoto	Bark Bark	Malaria Herpes zoster	Drinking Rubbing
	CM26	*Acacia hockii *De Willd.					
	CM49	*A. erioloba*,					
	CM61	*A. erubescens*,					
	CM69	*A. nigrescens*					

Moracea	CM25	*Ficus exasperate*	Fig Tree	Mukwiyu	Bark, Roots,	Lack of appetite Malaria	Drinking Drinking

	CM62	*F. thonningii*			Leaves Roots	Lack of appetite	Drinking

Moringaceae	CM42	*Moringa stenopetala L*	Phantom Tree	Moringa	Leaves	Vomiting, diarhoea	Drinking

Myrsinaceae	CM03	*Rapanea melanophloeus *(L.)	Cape Beech	Chisasa	Leaves Bark Seeds	Fungal infections Helminths	Rubbing Drinking

Myrtaceae	CM24				Leaves	Tuberculosis, Chronic diarrhoea, Coughing	Drinking
	CM63		Water Berry/pear	Mutoya	Bark	Chronic diarrhoea	
	CM42		Wild/Red syringa	Musheshe	Leaves/Bark	Herpes zoster, Herpes simplex, Skin	Rubbing
	CM48	*Psidium guajava *L. *Syzygium guineense *(Willd) DC *S. cordatum *Krauss *Burkea africana*				rashes	

Ochnaceae	CM14	*Lophira alata*		Muywe	Leaves Roots, Bark, Seeds	Malaria Malaria, Coughing, Gastrointestinal disorders	Drinking

Olacaceae	CM23	*Schrebera alata*	Large Sourplum	Mulutuluha Mukauke	Root	Skin rashes	Rubbing

	CM43	*Ximenia americana var. caffra *(Sond.) Engl.			Root bark	Candidiasis	Rubbing

Papilionaceae	CM16	*Dalbergia melanoxylon *Guill. & Perr.	Zebra wood	Mukelete	Leaves	Back and joint-aches oral candidiasis ulcer boils	Rubbing, Oral wash
	CM44	*Abrus precatorius *L.		Isunde	Leaves Roots, Bark		

Polygalaceae	CM22	*Securidaca longipedunculata *Fres.	Violet tree	Muinda	Leaves/Bark, Root	Cryptococcal meningitis, Oral candidiasis, Coughing	Drinking Oral wash Drinking

Rubiaceae	CM17	*Canthium zanzibarica *Klotzsch.		Mubilo	Bark, Root Leaves	Cryptococcal meningitis,	Drinking
	CM46 CM20	*Cathium burtti, Vangueria infausta*		Mubila		Oral candidiasis	Oral wash

Ruscaceae	CM21	*Sansevieria trifasciatai *Prain.		-	Leaves	Reduce pain and Inflammation	Rubbing

Tiliaceae	CM04 CM64	*Grewia bicolor *Juss. *G. avellana*,		Muzunzunyani	Leaves, Bark, Roots	Chronic diarrhoea	Drinking
	CM45	*G. falcistipula*,					
	CM70	*G. flava*,					
	CM19	*G. occidentalis*					

## Discussion

Our results show that traditional healers manage several AIDS-related conditions using a single plant species. This is not surprising given that a single plant species can contain several chemical compounds that can curtail several infections. On the other hand, different traditional healers also used more than one plant species to manage the same AIDS-related condition. Therefore, indigenous knowledge of medicinal use of plants is dynamic and varies according to healers, disease condition, and availability of plant species. Further, the results demonstrate that extracts from these plants could be administered as single or multi-plant remedies. This is important given the phenomenon of resistance to single plant use associated with HIV/AIDS-related infections, whereas resistance to multiple plant use is less likely to occur.

The survey revealed that Combretaceae (14%), Anacardiaceae (8%), Mimosaceae (8%), and Ebanaceae (7%) were the most predominant plant families used as ethnomedicines for AIDS-related conditions (Fig. 2). Although the active chemical compounds (and their modes of action) of the surveyed plants were largely unknown, it is plausible that the plant families contain bioactive secondary metabolites that work against AIDS-related infections. For example, previous studies reported that the family Anacardiaceae was rich in tannins, triterpenes, and flavonoids that help treat diarrhoea, dermal ulcers, general skin eruptions, and abdominal pains [8-12].

In Tanzania, Kisangau and co-workers found that the Anacardiaceae, Asteraceae, Capparaceae, Clusiaceae, Euphorbiaceae, Lamiaceae, Mimosaceae, Myrtaceae, Papillionaceae and Rubiaceae were predominantly used by traditional healers to manage HIV/AIDS opportunistic infections [13]. The families Myrtaceae and Lamiaceae were endowed with terpenoids, biological compounds that enhance and maintain body immunity [14]. Singh and others found that most plants used in the management of AIDS-related opportunistic infections contained flavonoids [15], a class of chemical compounds known to possess anti-oxidant properties that prevent free radical generation and tissue damage associated with the onset of AIDS. Antiviral activity of flavonoids was shown in animal models; hence drugs made from flavonoids could soon be accelerated towards human clinical trials.

In this study, the most plant parts used as ethnomedicines were leaves (33%), bark (32%), and roots (28%) (Fig. 3). The frequent harvesting of roots and barks may destroy the plants, and is therefore not advisable. To foster sustainability, traditional healers were encouraged to use plant leaves. For example, *Aloe zebrina *leaves were used to treat herpes zoster in Katima Mulilo, Namibia. In Tanzania, Kisangau and co-workers also reported the use of *Aloe *plants for the treatment of herpes zoster [13] whilst in Kiambu and Murang'a districts of Kenya, *Aloe *plants were used to treat malaria [16]. In Katima Mulilo, herpes zoster was generally treated with several plants, including *Rhus natalensis*, *Annona senegalensis*, *Capparis tomentosa*, *Garcinia buchananii*, and *Syzygium guineense*. These findings conform very well with those of traditional healers in the Bukoba rural district of Tanzania where the same plants were used in the treatment of herpes zoster [13].

Diarrhoea is one of the most prevalent opportunistic infections during AIDS. Our study documented 21 different plant species used to manage diarrhoea in Katima Mulilo (Table 1). Most of these plants have also been reported to treat chronic diarrhoea and dysentery in other studies: *Schlerocarya birrea *[17]; *Rhus tenuinervis*, *Capparis tomentosa*, *Burkea africana*, *Kigella Africana*, *Terminalia sericea*, *Combretum apiculatum *and *Hibscus fuscus *and *H. sabdariffa *[13]; *Adansonia digitata *[18]; *Combretum glutinosum *[19]; *Croton lechleri *[20]; *Pterocarpus erinaceus *[18]; and *Moringa stenopetala *[21].

A number of plant species were used to treat oral candidiasis in Katima Mulilo: *Sclerocarya birrea*, *Lannea stuhlmannii*, *Capparia tomentosa*, *Antidesma venosum*, *Ximenia Americana*, *Abrus precatorius*, and *Vangueria infausta*. Elsewhere, it was also revealed that *Dichrostachys cinerea*, *Lannea stuhlmannii*, and *Sclerocarya birrea *had anti-Candida activity [17]. *Antidesma venosum *[20], *Ximenia Americana *[22], and *Abrus precatorius *[18] were also used as ethnomedicines for oral candidiasis. While *Ximenia americana *was further used to treat skin rashes and toothache in Katima Mulilo. Vermani and Garg [23] reported that the same plant was used to treat contagious diseases, stomach complaints and worm infestations in India.

Malaria, a common condition among AIDS-patients in Katima Mulilo, is managed with 17 different plant species. Some of these plants were found to treat malaria in other studies conducted elsewhere: *Xylopia spp*.[24], *Adansonia digitata *and *Lophira alata *[18], *Combretum glutinosum *and *Guibourtia tessmannii*, *Ficus exasperata *and *Ficus thonningii *[19], *Cucumis aculeatus *[16], *Diospyros spp*.[25,26], and *Albizia anthelmintica *[27]. In Tanzania, *Capparis erthrocarpis *was also used to tuberculosis [13], while skin rashes were treated with *Garcinia buchananni *[13]. In other studies, *Commiphora Africana *was used to treat swollen pancreas [28], while *Rapanea melanophloeus *treated fungal infections [29] and roundworms [22]. Recently, *Sansevieria bicolor *was reportedly used to treat pain and inflammation [30]. Two fig tree species (*Ficus exasperate *and *F. thonningii*) were variously reported to treat malaria and lack of appetite [19,20,31]. Other reports indicated that *Dalbergia melanoxylon *leaves reduced back- and joint-aches [27] while *Moringa stenopetala *reduced vomiting and diarrhoea [21].

Although the use of ethnomedicines to manage HIV/AIDS has recently gained public interest in Namibia, harmonization with official HIV/AIDS policy remains a sensitive and contentious issue [6]. It is sensitive because traditional medicines can easily become a scapegoat for denial and inertia to roll-out ART as was the case during President Thabo Mbeki's South Africa [6]. It is also contentious because in many resource-poor settings in Sub-Saharan Africa, government-sponsored ART programmes discourage the use of traditional medicines, fearing that the efficacy of antiretroviral drugs may be inhibited by traditional medicines, or that their interactions could lead to toxicity [32]. Reliance on traditional medicines can also lead to a discontinuation of ART therapy [33]. Thus many African governments including Namibia still have contradictory attitudes towards traditional medicines for AIDS, discouraging it within ART programmes, and supporting it within their initiatives of public health and primary health care [6].

Despite this contradictory scenario, indigenous plants and mushrooms have been embraced as potential reservoirs that may contain a large repertoire of novel anti-HIV active compounds. Unfortunately, anti-HIV active compounds from these natural products have not been isolated. The Namibian government has set up an Indigenous Plant Task Team (IPTT), and through the New Partnership for Africa's Development/Southern African Network for Biosciences (NEPAD/SANBio), the University of Namibia (UNAM) was nominated as the focal point to spearhead the country's participation in this sub-regional project whose aim is to isolate anti-HIV active compounds from indigenous plants. UNAM scientists to be resident at the Council for Scientific and Industrial Research (CSIR), Pretoria, South Africa will carry out isolation of anti-HIV active compounds from four selected Namibian plants. Further, the results of this study form part of the preliminary efforts to set up a Namibian pharmacopeia of indigenous plants used to treat HIV/AIDS and related opportunistic infections. This will help preserve knowledge of prospective indigenous plants with novel anti-HIV activity. A database of anti-HIV plants is important given that most healers are old and may die with their libraries of knowledge.

The current collaboration will also enhance local skills and drugs development. However, a few challenges such as intellectual property rights and trans-boundary shipment of plants remain unresolved. Resolution of these issues is being undermined by the lack of national legislation relating to indigenous plants and knowledge, genetic resources, access and benefit sharing (ABS). Government has instituted the National Biodiversity Programme (NBF), the IPTT, and the Interim Plant Bioprospecting Council (IPBC), mandated by Cabinet to formulate policies and legislation to regulate these matters. A Bill on ABS has been drafted but is yet to be enacted into law because technical questions relating to its implementation remain unanswered [34].

## Conclusion

Traditional healers' indigenous knowledge can help pinpoint medicinal plants used to manage HIV/AIDS. In this study, 28 plant families consisting of 72 species were used as ethnomedicines for HIV/AIDS-related opportunistic infections in Katima Mulilo, Caprivi region, Namibia. These plants treated conditions such as herpes zoster, diarrhoea, malaria, coughing, tuberculosis, and meningitis. Since traditional healers harvest roots and barks of these medicinal plants, there is need to educate them about the looming danger of wiping out some of the over-exploited plant species. Further research is also needed to isolate the plants' active chemical compounds, in addition to deciphering their modes of action.

## Competing interests

The authors declare that they have no competing interests.

## Authors' contributions

KC developed the research study, spearheaded the research project, led collaboration with traditional healers, collected data and wrote the manuscript. MH collaborated with traditional healers, collected data and wrote the manuscript. KC and MH read and approved final manuscript.

All authors have read and approved the final manuscript.

## References

[B1] Government of the Republic of Namibia Report of the 2008 national HIV sentinel survey: HIV prevalence rate in pregnant women, biannual screening 1992-2008, Namibia 2008 Ministry of Health and Social Services: Windhoek, Namibia

[B2] UNAIDS/UNGASS Namibia 2010 country progress report; reporting period 2008-2009 2010 Ministry of Health and Social Services: Windhoek, Namibia

[B3] WHO Namibia country summary profile for HIV/AIDS treatment scale-up 2005 http://who.int/3by5/support/june2005_nam.pdf

[B4] Government of the Republic of Namibia HIV/AIDS in Namibia: behavioural and contextual factors driving the epidemic 2008 Ministry of Health and Social Services/USAIDS/MEASURE Evaluation: Windhoek, Namibia

[B5] Government of the Republic of Namibia The National Strategic Plan on HIV/AIDS. Third medium term plan 2004-2009 2002 Directorate, Special Programmes, Ministry of Health and Social Services: Windhoek, Namibia

[B6] Chinsembu KC Model and experiences of initiating collaboration with traditional healers in validation of ethnomedicines for HIV/AIDS treatment in Namibia J Ethnobio Ethnomed 2009 5 30 10.1186/1746-4269-5-30 PMC2771007 19852791

[B7] Government of the Republic of Namibia Caprivi region livelihood baseline profile- lowland maize and livestock zone 2009 Office of the Prime Minister- Directorate Emergency Management: Windhoek, Namibia

[B8] Rwagabo PC Umuhengerin, a new antimicrobially active flavonoid from Lantana trifolia J Nat Prod 1998 51 5 966 968 10.1021/np50059a026 3204384

[B9] Kokwaro JO Medicinal plants of East Africa 1993 2 Kenya Literature Bureau: Nairobi, Kenya

[B10] Mona H Health benefits of *Morinda citrifolia* Consumer Health 1997 20 12

[B11] Okoli AS Okeke MI Iroegbu CU Ebo PU Antibacterial activity of *Harungana madagascariensis *leaf extracts Phytother Res 2002 16 2 174 179 10.1002/ptr.991 11933123

[B12] Repetto MG Liesuy SF Antioxidant properties of natural compounds used in popular medicine for gastric ulcers Braz J Med Biological Res 2002 35 5 523 534 10.1590/s0100-879x2002000500003 12011936

[B13] Kisangau DP Lyaruu HVM Hosea KM Joseph CC Use of traditional medicines in the management of HIV/AIDS opportunistic infections in Tanzania: a case in the Bukoba rural district J Ethnobio Ethnomed 2007 3 29 10.1186/1746-4269-3-29 PMC1941724 17623081

[B14] Wagner KH Emadfa I Biological relevance of terpenoids: overview focussing on mono-, di- and tetraterpenes Annals of Nutr Metabol 2003 47 95 106 10.1159/000070030 12743459

[B15] Singh IP Bharate SB Bhutani KK Anti-HIV natural products Cur Science 2005 89 2 269 289

[B16] Njoroge GN Bussmann RW Diversity and utilization of antimalarial ethnophytotherapeutic remedies among the Kikuyus (Central Kenya) J Ethnobio Ethnomed 2006 2 8 10.1186/1746-4269-2-8 PMC1397805 16451716

[B17] Runyoro DKB Matee MIN Ngassapa OD Joseph CC Mbwambo ZH Screening of Tanzanian medicinal plants for anti-Candida activity BMC Compl Alter Med 2006 6 11 10.1186/1472-6882-6-11 PMC1481531 16571139

[B18] Kayode J Conservation of indigenous medicinal botanicals in Ekiti State, Nigeria J Zhejiang Univ SCIENCE B 2006 7 9 713 718 10.1631/jzus.2006.B0713 PMC1559802 16909472

[B19] Titanji VPK Zofou D Ngemenya MN The antimalarial potential of medicinal plants used for the treatment of malaria in Cameroonian folk medicine Afr J Trad CAM 2008 5 3 302 321 PMC2816552 20161952

[B20] Jernigan KA Barking up the same tree: a comparison of ethnomedicine and canine ethnoveterinary medicine among the Aguaruna J Ethnobio Ethnomed 2009 5 33 10.1186/1746-4269-5-33 PMC2777850 19903346

[B21] Mesfin F Demissew S Teklehaymanot T An ethnobotanical study of medicinal plants in Wonago Woreda, SNNPR, Ethiopia J Ethnobio Ethnomed 2009 5 28 10.1186/1746-4269-5-28 PMC2769162 19821994

[B22] Nanyingi MO Mbaria JM Lanyasunya AL Wagate CG Koros KB Kaburia HF Munenge RW Ogara WO Ethnopharmacological survey of Samburu district, Kenya J Ethnobio Ethnomed 2008 4 14 10.1186/1746-4269-4-14 PMC2412850 18498665

[B23] Vermani K Garg S Herbal medicines for sexually transmitted diseases and AIDS J Ethnopharmacol 2001 80 49 66 10.1016/S0378-8741(02)00009-0 11891087

[B24] Botsaris AS Plants used traditionally to treat malaria in Brazil: the archives of Flora Medicinal J Ethnobio Ethnomed 2007 3 18 10.1186/1746-4269-3-18 PMC1891273 17472740

[B25] Kantamreddi VSS Wright CW Investigation of Indian Diospyros Species for Antiplasmodial Properties Compl Alter Med 2008 5 2 187 190 10.1093/ecam/nem019 PMC2396478 18604258

[B26] Muazu J Kaita AH A review of traditional plants used in the treatment of epilepsy amongst the Hausa/Fulani tribes of northern Nigeria Afr J Trad CAM 2008 5 4 387 390 10.4314/ajtcam.v5i4.31294 PMC2816574 20161961

[B27] Kareru PG Gachanja AN Keriko JM Kenji GM Antimicrobial activity of some medicinal plants used by herbalists in eastern province, Kenya Afr J Trad CAM 2008 5 1 51 55 10.4314/ajtcam.v5i1.31256 PMC2816592 20162055

[B28] Otieno JN Hosea KMM Lyaruu HV Mahunnah RLA Multi-plant or single-plant extracts, which is the most effective for local healing in Tanzania? Afr J Trad CAM 2008 5 2 165 172 10.4314/ajtcam.v5i2.31269 PMC2816543 20161933

[B29] Moshi MJ van den Beukel CJP Hamza OJM Mbwambo ZH Nondo ROS Masimba PJ Matee MIN Kapingu MC Mikx F Verweij PE van der Ven AJAM Brine shrimp toxicity evaluation of some Tanzanian plants used traditionally for the treatment of fungal infections Afr J Trad CAM 2007 4 2 219 225 10.4314/ajtcam.v4i2.31211 PMC2816448 20162095

[B30] Samuel AJSJ Kalusalingam A Chellappan DK Gopinath R Radhamani S Husain HA Muruganandham V Promwichit P Ethnomedical survey of plants used by the Orang Asli in Kampung Bawong, Perak, West Malaysia J Ethnobio Ethnomed 2010 6 5 1 6 10.1186/1746-4269-6-5 PMC2843656 20137098

[B31] Techlehaymanot T Giday M Ethnobotanical study of medicinal plants used by people in Zegie Peninsula, northwestern Ethiopia J Ethnobio Ethnomed 2007 3 12 10.1186/1746-4269-3-12 PMC1852296 17355645

[B32] Hardon A Desclaux A Egrot M Simon E Micollier E Kyakuwa M Alternative medicines for AIDS in resource-poor settings: insights from exploratory anthropological studies in Asia and Africa J Ethnobio Ethnomed 2008 4 16 doi:10.1186/1746-4269-4-16 10.1186/1746-4269-4-16 PMC2503967 18616794

[B33] Langlois-Klassen D Kipp W Jhangri GS Rubaale T Use of traditional herbal medicine by AIDS patients in Kabarole District, western Uganda Am J Trop Med Hyg 2007 77 757 763 17978084

[B34] Du Plessis P Indigenous knowledge and biotrade Presentation at the National Biosciences Forum and validation of traditional medicines workshop, Safari Hotel, Windhoek, November 20, 2007

